# Nontraditional Cardiovascular Biomarkers and Risk Factors: Rationale and Future Perspectives

**DOI:** 10.3390/biom8020040

**Published:** 2018-06-15

**Authors:** Irene Traghella, Francesca Mastorci, Pepe Alessia, Alessandro Pingitore, Cristina Vassalle

**Affiliations:** 1 Fondazione G. Monasterio CNR-Regione Toscana and Institute of Clinical Physiology, CNR San Cataldo Research Area, via Moruzzi, 1, 56124 Pisa, Italy; itraghella@ftgm.it (I.T.); alessia.pepe@ftgm.it (P.A.); 2 Institute of Clinical Physiology, CNR San Cataldo Research Area, via Moruzzi, 1, 56124 Pisa, Italy; mastorcif@ifc.cnr.it (F.M.); pingi@ifc.cnr.it (A.P.)

**Keywords:** cardiovascular risk, biomarker, vitamin D, hepatitis C virus, bone turnover biomarkers, psycho-emotional factors

## Abstract

The primary prevention of cardiovascular (CV) disease depends on the capacity to identify subjects at higher risk long before the occurrence of CV clinical manifestations. Traditional risk factors do not cover fully prediction of individual risk. Moreover, there is an area of gray for patients at intermediate CV risk, which offers wide margins of improvement. These observations highlight the need for new additive tools for a more accurate risk stratification. An increasing number of candidate biomarkers have been identified to predict CV risk and events, although they generally give only a moderate increase when added to currently available predictive scores. The approach utilizing a relative small number of biomarkers in multiple combinations, but only weakly related to each other or unrelated, thus belonging to independent-pathways, and so able to catch the multidimensional characteristic of atherosclerosis, appears promising. We discuss vitamin D and bone turnover biomarkers, hepatitis C virus, and psycho-emotional factors that may reflect alternative pathways over those generally considered for atherosclerosis (e.g., aspects directly related to inflammation and thrombosis). These new biomarkers could facilitate a more accurate assessment of CV risk stratification if incorporated in the current risk assessment algorithms.

## 1. Introduction

A biomarker is defined as a measurable indicator of physiological biologic processes, pathologic processes, or responses to behavioral or therapeutic interventions; therefore, as the main interest of research efforts remains to increase our knowledge about the causes of diseases, the identification of new biomarkers and assessment of their physiopathological value is crucial [1]. Traditional available algorithms for the estimation of cardiovascular (CV) risk are widely known and used in the clinical practice [2]. These tools (e.g., the Framingham heart studyrisk score, FRS) stratify patients into low-, intermediate-, and high-risk categories. Unfortunately, the assessment of CV risk through these traditional tools is not optimal for all categories of patients [3]. In particular, there is a great percentage of subjects which incur in CV events without presenting any traditional risk factors, as well as there is an area of gray for groups of patients at intermediate risk, which offer wider margins of improvement. It is precisely in these cohorts of subjects that would be desirable a further classification towards a lower or higher risk, introducing further targeted and reliable biomarkers to improve risk stratification, or identifying subclinical atherosclerosis and thus allowing more effective prevention and treatment strategies.

Manifest CV disease is preceded by a long period of asymptomaticity, and as biomarkers may reflect a wide number of pathological events involved in the progression of atherosclerosis, they may be informative of different phases of the disease. In the CV field, besides environmental risk factors (e.g., smoking habit and diet), we potentially have at our disposal biomarkers of different nature, including genetic, circulating, and imaging biomarkers [4,5,6,7]. Among them, many resulted associated to different phases of CV disease and prognosis [4,5,6,7,8,9]. Genetic biomarkers explain the potential of heritable profiling for CV disorders, providing a tool for early diagnosis or prediction phases in each individual subject [4,5]. Biochemical biomarkers, measurable in blood or urine, can be an expression both of early processes in the activation and subclinical phases or of stages when the damage is clearly established [6]. Moreover, different imaging techniques have been developed to characterize plaques from many different perspectives (e.g., plaque measurement, plaque component quantifying, visualization, and vessel functions) [7,8]. However, their single additional value for risk assessment, over the one obtained by calculation with traditional algorithms, is generally found rather limited [10]. Due to the poor individual predictive performance of each biochemical parameter and to the generally limited differential potential capacity of each circulating biomarker to predict different types of events and to mark different pathogenetic phases in the development of vascular disease, it has been proposed that the use of more circulating biomarkers in specific panels may be more effective in order to assess CV risk estimation [10]. However, at the moment, even this “multimarker approach” has not given the expected results and at most moderately improves risk prediction [10]. Generally, biomarkers belonging to known pathogenic pathways, mostly including inflammation and thrombosis-related parameter, are included in these panels. However, discrimination capacity and performance of this approach can be improved exploiting the low degree of correlation between biomarkers, and the multidimensional characteristic of atherosclerosis [3]. In fact, when several biomarkers of a known biological pathway are measured, this can lead to a lack of a substantial gain. Instead, assessment of global risk may require the integration of multiple biomarkers and the evaluation of biomarkers belonging to independent-pathways. In this context, a panel of nine biomarkers, including vitamin D (25(OH)D), and representing different pathophysiological pathways (heart function: heart rate, left ventricular ejection fraction, and N-terminal pro b-type natriuretic peptide (NTproBNP); kidney function: cystatin C; glucose metabolism: glycated hemoglobin (HbAc1); blood pressure: renin, and calcium homeostasis) have been evaluated to generate a new risk score based on the Vienna and Ludwigshafen CAD (VILCAD) score (originally composed by clinical and biochemical variables, and focused on risk stratification in patients with stable CAD) [11]. A simplified score—including male gender, age, heart rate, left ventricular ejection fraction, NTproBNP, HbA1c, renin, 25(OH)D, and cystatin—was demonstrated effective to improve reclassification correctly downgrading the risk level of event-free individuals, with an even larger percentage of patients with events who were correctly reclassified [11]. In particular, almost half of patients, classified as low risk by the original VILCAD score, were upgraded to moderate risk by the biomarker score, and about one third of patients originally classified as moderate risk were upgraded to the high-risk group [11]. Other data indicate that low 25(OH)D (<20 ng/mL) (odds ratio, OR, (95% confidence intervals, CI)) (5 (1.4–18) *p* < 0.05) and elevated osteocalcin (OC) (bone turnover biomarker) (>75^th^ percentile-16.6 ng/mL) (6.7 (1.9–23.8) *p* < 0.01) were found to be significant FRS predictors, while subjects with elevated OC and/or bone alkaline phosphatase (bone turnover biomarker: BALP; >75^th^ percentile-9.8 μg/L) showed a higher CV risk as estimated by PROCAM score (3.6 (1.2–10.7) *p* < 0.05) [12]. Very recently, the additive role of OC over that obtained from the atherosclerotic cardiovascular disease (ASCVD) risk score to identifying subclinical atherosclerosis (represented by the carotid intima-media thickness, C-IMT) was evaluated [13]. Levels of OC resulted independently and inversely associated with elevated C-IMT after adjusting for the 10-year ASCVD risk score (*p* = 0.004), this negative relationship remaining statistically significant more in the subset of subjects with a moderate-to-high ASCVD risk [13].

At the moment, the additive value of new recently proposed CV risk biomarkers over available risk scores, as well as the composition of possible multimarker panels is far to be evaluated and widely shared. However, among the large number of recently proposed innovative biomarkers and CV risk factors, there are some (vitamin D-hormone, bone turnover biomarkers-bone, hepatitis C virus (HCV)-infectious agents, and psycho-emotional factors) which appear promising in the opinion of the authors and below discussed. These biomarkers relate to atherosclerosis processes reflecting different pathophysiological aspects, and could facilitate a more accurate CV risk assessment if evaluated and incorporated in the current risk assessment algorithms.

## 2. Vitamin D and Biomarkers of Bone Turnover

### 2.1. Vitamin D

Vitamin D is a hormone, responsible for correct bone mineralization and as such involved in intestinal absorption of calcium, magnesium, and phosphate [14]. Through ultraviolet B (UVB) rays, sunlight, more than diet, is the main source of vitamin D, providing the conversion of 7-dehydrocholesterol to previtamin D3, then isomerized to vitamin D3 (cholecalciferol) [14]. Cholecalciferol, bounded to vitamin D binding protein (DBP) in blood, undergoes two hydroxylation, the first in the liver into 25(OH)D, the second in the kidney into 1,25(OH)2D, the active hormone, by 1-α hydroxylase [14]. Behind the well-known skeletal actions, there are other many different conditions which can be affected by vitamin D levels. Among them, the relationship with CV disease is evidenced by the fact that vitamin D receptor has been identified throughout the CV system [15].

Although 1,25(OH)_2_D is the active metabolite, the total form (25(OH)D) is more appropriate to assess vitamin D status. In fact, as 1,25(OH)_2_D value is finely regulated, a low vitamin D status may induce 1,25(OH)_2_D parathyroid hormone (PTH)-related increase following secondary hyperparathyroidism, with normal or even elevated 1,25(OH)_2_D levels [14]. Generally, vitamin D insufficiency is considered associated to 25(OH)D levels between 21 and 29 ng/mL (corresponding to 52.5–72.5 nmol/L), deficiency corresponds to 25(OH)D levels less than 20 ng/mL, and severe deficiency to 25(OH)D less than 10 ng/mL (<25 nmol/L) [14]. According to this definition, up to 80–100% of the entire worldwide population may have inadequate serum 25(OH)D [16]. In addition, it has been estimated that around half of children and adolescents are at high risk for vitamin D deficiency and insufficiency worldwide [17]. These observations render necessary the significance of these findings in terms of extra-bone disease risk. In fact, optimal vitamin D concentration has been estimated from a minimum threshold of 30 ng/mL [18]. This value, assessed for bone health, is based on the fact that at 30 ng/mL or above vitamin D is not a limiting factor for calcium absorption, and that PTH levels are minimized at this level [19,20]. However, a threshold or multiple thresholds for extra-bone conditions are yet to be defined [14]. Specifically, whether 20 and 30 ng/mL thresholds may be equally effective when applied to the CV risk remains to be further evaluated.

In any case, many different direct and indirect actions by which vitamin D could affects the CV system have been identified [21]. Vitamin D has direct effects on calcium influx and myocyte contractility [22]. In addition, vitamin D has antioxidant properties and affects angiogenesis and platelet aggregation, inflammatory parameters, and endothelial function [23,24]. Many CV risk factors (e.g., obesity, sedentary lifestyle, hypertension, and diabetes) and CV adverse events are associated hypovitaminosis D [12,21,24]. Accordingly, a recent meta-analysis (19 independent studies with 6123 CV disease cases in 65,994 participants) evidenced a significant inverse correlation between vitamin D and CV risk [25]. In particular, the comparison of the lowest with the highest 25(OH)D categories evidenced a pooled relative risk of 1.52 (95% confidence interval, 1.30–1.77) for total CV disease; 1.42:1.19–1.71 for CV mortality, 1.38:1.21–1.57 for coronary heart disease, and 1.64:1.27–2.10 for stroke [25].

Meta-analyses of observational studies confirmed the key role of low vitamin D for CV risk and CV and overall mortality and events [26,27,28]. In particular, a recent meta-analysis including 180,667 participants in perspective studies (27 independent studies for total CV events and 17 independent studies for CV mortality) confirmed that 25(OH)D concentration was inversely associated with total CV disease events and CV mortality (pooled relative risks (RRs) per 10 ng/mL 25(OH)D increment corresponding to 0.90 (95% CI: 0.86, 0.94) for total CV events and 0.88 (95% CI: 0.80, 0.96) for CV mortality [28]. However, the effect of vitamin D supplementation on CV risk reduction is still unclear, as antioxidant trials in the CV field have previously been reported to be non-effective or may even increase cardiovascular risk, as shown for example for vitamin E [29]. However, there are many pitfalls for the lack of effects of exogenous antioxidant supplementation, that may be related to vitamin doses (too high/too low), the duration of supplementation (long-term antioxidant supplementation effects on health are unknown) and interaction with other exogenous antioxidants given in a combined antioxidant supplementation or antioxidants contained in foods. Indeed, experimental data suggested that high antioxidant levels may induce adverse pro-oxidant effects. Thus, it would be necessary for an estimation of baseline individual’s oxidative stress status before deciding on about possible antioxidant supplementation, measure that generally is not done in majority of studies. This point is important, as subjects with higher oxidative stress likely benefit more of these vitamin (antioxidant) supplementation. Moreover, it is also important to consider that oxidative stimuli can be beneficial, according to hormesis theory (biological beneficial response from exposure to low doses of an agent that is instead dangerous when present at high levels), whereas elevated antioxidant supplementation may reduce hormesis. All these aspects may be taken in account in future trials. In this context, the supplementation with vitamin D combined with calcium appears more effective to improve overall mortality than vitamin D alone [30]. However, in The Women’s Health Initiative (WHI) study, vitamin D3 (400 international units (IU)/day) and calcium supplementation (1000 mg of elemental calcium daily) did not affect CV events during 7 years of follow-up [31]. It is noteworthy that the dose used in the WHI was low. In this context, another meta-analysis suggests that vitamin D supplementation at moderate-to-high doses (approximately 1000 IU/day) may reduce CV risk [32]. Moreover, baseline vitamin D levels were not performed in majority of available studies, whereas it is conceivable that subjects with lower vitamin D levels benefit more of vitamin D supplementation. Conversely, whether the strength of the associations of vitamin D and CV risk and events is well recognized, a causal role is harder to prove [21,24]. As low 25(OH)D is related to CV risk factors, the possibility exists that low vitamin D in CV disease is only an epiphenomenon. As an example, vitamin D may be lower in obese subjects, a well-known CV risk factor, because of the lipophilic nature of vitamin D and its sequestration in the fat [33]. Moreover, elderly subjects may also have lower 25(OH)D levels simply because they spent less time outdoors [34].

These recent years of high-throughput technological evolution, paved the way toward a new era of new technological developments in research, improving our knowledge of pathways relevant to health and diseases. Interestingly, proteomic analysis aimed to identify proteins expressed in the plasma of survivors of myocardial infarction and possible correlations between expression of some proteins and the severity of coronary artery disease (CAD), evidenced decreased levels of DBP in CAD patients, which significantly correlated with the number of affected coronary arteries, and to disease severity [35]. This finding was also confirmed by enzyme-linked immunosorbent assay test [35]. Nonetheless, in serum samples of patients with ST-elevation myocardial infarction (STEMI) the proteomic approach identified increased levels of DBP when compared to control donors, confirmed by the Western blot analysis [36]. These findings were confirmed by those recently published on the proteome profile changes after acute myocardial infarction (AMI) in an experimental model, where the expression levels of DBP and vitamin D receptor (VDR) were found increased in both left ventricular tissue of AMI mouse model, suggesting that DBP might be involved in left ventricular remodeling, and H9C2 cells, promoting cardiomyocyte apoptosis, after hypoxia [37].

### 2.2. Osteocalcin

Osteocalcin, produced by osteoblasts, is traditionally considered a marker of bone formation, involved in the process of mineralization. However, OC has been also associated to vascular calcification and atherosclerosis. Moreover, analysis of transgenic mouse models, including OC^−/−^, Esp^−/−^, and FoxO1_ob_^−/−^, revealed a systemic effect of OC on glucose metabolism, with effects ranging from insulin production and secretion from the pancreas to secretion of adiponectin, an adipokine which enhances insulin sensitivity through activation of adenosine monophosphate (AMP)-activated protein kinase [38,39]. In particular, OC may locally affect the metabolic state of cells, (e.g., chondrocytes and vascular smooth muscle cells, VSMCs), and induce their differentiation and mineralization, through activation of hypoxia-inducible factor 1 α (HIF-1 α) which promotes glucose uptake and modulate its metabolism [40]. There is probably a mutual relationship between OC and insulin, which likely act on each other in an interconnected network. In fact, insulin increases OC production and activation, which in turn stimulates pancreatic β-cell proliferation and insulin secretion, adiponectin production, and improves insulin sensitivity. In this context, some hormones, such as leptin and glucocorticoids, negatively regulate OC activity by suppressing osteoblast function, osteocalcin secretion, and energy expenditure [41,42]. Accordingly, higher OC has been inversely found associated with body mass index, waist circumference, glucose, insulin, and homeostasis model assessment-estimated insulin resistance (HOMA-IR) and with lower prevalence of diabetes [43,44,45,46]. In a recent prospective study conducted on a Japanese cohort of postmenopausal women, lower OC levels resulted associated with future development of type 2 diabetes (T2D) independently of conventional risk factors [47].

Low OC was inversely associated with subclinical atherosclerosis (brachial-ankle pulse wave velocity, carotid intima-media thickness), presence of atherosclerotic plaques in adult subjects and T2D patients, and also inversely correlated with C reactive protein [48,49,50]. Moreover, a relationship between lower OC and presence and extent of CAD as well as glucose metabolic biomarkers (fasting and post load-2 hour-glucose and HbA1c) was observed in a large Chinese cohort of patients who underwent coronary angiography (243 with CAD and 218 without CAD) [51].

Interestingly, a recent study evidenced that differences in the relationship between OC and metabolic biomarkers of glucose metabolism and β-cells function may exist according the membership to different ethnicities [52]. This fact might explain why other authors have reported different results, including a higher prevalence of carotid atherosclerosis in healthy postmenopausal women with elevated OC and low bone mineral density, or higher CV risk (as estimated by FRS, and PROCAM scores) associated to higher OC levels in adults [12,53].

Interestingly, OC could find application in elusive clinical settings, such as in premature AMI (with event occurring ≤40 years of age) where levels of OC appear significantly lower as compared to controls (medians: interquartile ranges; 16.7:12.4–21.5 ng/mL vs., 21:15.2–27.6 ng/mL) [54].

The assessment of OC in humans included both the carboxylated (cOC) and uncarboxylated forms (ucOC), whereas ucOC appeared associated to higher HOMA-B% levels, and thus to β-cell function, while cOC was associated to lower HOMA-IR values, more representative of insulin resistance [55]. Interestingly, some data suggested that values lower than a ucOC/cOC index cutoff, corresponding to 0.3, can be associated with markers of poor metabolic control in T2D [56].

As it concerns vascular calcification, it has been proven that OC is expressed by atherosclerotic plaques and VSMCs, which testify the regulatory role of this protein in atherosclerotic calcification [57]. Moreover, other experimental data further showed that OC appeared produced by calcifying VSMCs, whereas treatment with bisphosphonates simultaneously decreased vascular calcification and lowered serum OC levels [58].

It is also known that patients with coronary atherosclerosis may express OC in a higher percentage of circulating endothelial progenitor cells (EPCs)—which stimulate endothelial cell proliferation and migration and promote new vessel formation—when compared with controls with a normal endothelial function [59]. Interestingly, other data suggested that early and highly active EPCs, carrying the osteoblastic marker OC, are increased in patients with myocardial infarction history, when compared with patients with stable CAD or CV-RFs without established CAD [60]. These findings may suggest that this subset of EPCs could affect vascular calcification and repair, and could help as additive tool to further stratify more stable or unstable atherosclerotic patients [60]. Moreover, they can potentially serve as a marker for adverse CAD risk and prognosis, as a very high numbers of early circulating OC positive EPCs were closely related with CAD severity, and tended to be associated with the risk of all-cause mortality [61].

A recent review and meta-analysis (26 positive, 17 negative, and 29 neutral relationships for association between OC in blood, presence of OC-positive cells, or OC histological staining and extent of calcification or atherosclerosis) failed to find a clear and definitive association between OC and vascular calcification or atherosclerosis, although a consistent positive correlation was evidenced between the presence of OC-positive cells and histological staining with calcification and atherosclerosis [62]. Interestingly, also in this case ethnic differences may greatly affect these results, as 37% of Asian studies reported negative relationships between OC and calcification or atherosclerosis, compared to 6% of European studies, suggesting genetic predisposition-related differences [62].

### 2.3. Other Bone Turnover Biomarkers

Bone has emerged as a critical regulator of glucose and energy metabolism and also other bone proteins than OC has been shown to be centrally involved as potential pathophysiologic culprit as well as possible targets of diabetic complications. Procollagen type I N-propeptide (PINP) and procollagen type I C-propeptide (PICP) represent the extension peptides at the N- and C- end of the procollagen molecule, which are cleaved during type I collagen synthesis, and released into the circulation [21]. Conversely, the N- and C-terminal crosslinking telopeptides of type I collagen (NTx and CTx, respectively) are considered bone resorption indices, and represent proteolytic fragments of bone collagen matrix [21].

These bone turnover biomarkers have been essentially associated to cardio-metabolic risk. Some data suggested that in 32 years old men with metabolic syndrome (MetS), P1NP, together with OC resulted lower compared to healthy men, and inversely correlated with insulin and glucose, although these relationships lost significance in a fully adjusted model [63]. In premenopausal women with T2D, P1NP, and CTx are lower when compared with non-T2D controls (median, interquartile range: 29.9 ng/mL, 24.7–41.8 ng/mL vs., 37.3 ng/mL, 30.8–47.3 ng/mL, *p* < 0.01, and 0.161 ng/mL, 0.106–0.227 ng/mL vs., 0.202 ng/mL, 0.166–0.271 ng/mL, *p* <  0.01, respectively) [64]. Also in elderly, postmenopausal diabetic women levels of bone turnover biomarkers, including CTx and OC, were reduced with respect to subjects without T2D [65]. Moreover, also in T2D male patients, CTx levels were negatively associated with HbA1c (*r* = −0.41, *p* < 0.05) [66]. Interestingly, the adoption of a Mediterranean diet enriched with virgin olive oil for 2 years is able to modulate a P1NP and OC levels, both biomarkers increased significantly, suggesting a protective effect on bone [67]. Moreover, weight-loss has been generally associated with increase in bone formation as well as resorption biomarkers [68,69]. However, very recent data, obtained in a mouse experimental model, evidenced high-carbohydrate high-fat (HCHF) diet caused a significant increase in CTx level [70]. This finding suggests that MetS induced by HCHF diet adversely affects bone due to an imbalance between osteoblastic and osteoclastic activities, reflected by the elevation of bone resorption biomarker [70].

As it concerns hypercholesterolemia, experimental and human studies evidenced that this condition generally increases bone turnover biomarkers, which showed negative correlation with high density lipoprotein cholesterol, and a significantly positive correlation with total cholesterol and low density lipoprotein cholesterol [71,72]. In particular, some experimental data, showed that high-fat diet increased CTx in hyperlipidemic mice [73].

These biomarkers can also affect negatively CV prognosis. As an example, in a large cohort of old men (3384 men, 70–89 years, followed for 7.0 years), elevated levels of P1NP and the ucOC/OC (expressed as %) were associated with higher incidence of MI, although not of stroke, whereas CTx was not associated with incident myocardial infarction or stroke [74]. In a prospective study of 1112 frail elderly subjects (79% female; mean age, 86 years) CTx was significantly associated with deaths from cardiac causes (hazard ratio (HR) = 1.78: 95% CI: 1.27–2.50; *p* < 0.001) [75]. In another study, conducted on 986 women aged 65 (58–72) years referred to coronary angiography, the highest CTx quartile was associated with an increased risk of all-cause and cardiovascular mortality [76]. The lowest 25(OH)D quartile was associated with a trend towards increased risk of noncardiovascular mortality in multivariate analysis, while OC quartile 2 and 3 were significantly associated with lower risk of noncardiovascular mortality [76].

Bone alkaline phosphatase (BALP) is important for the mineralization of bone and represents a useful biomarker of bone formation. In pre-dialysis chronic kidney disease (CKD) patients, arterial stiffness has been found correlated with BALP, which might represent a possible clinical predictor of CV disease in CKD [77].

Thus, in view of these controversial findings (bone turnover biomarker reduction or increase) in different clinical CV setting, more studies are needed to better understand the complex, interconnected network between bone and cardio-metabolic risk and their reciprocal relationship.

## 3. HCV and Infection

Although the relationship between atherosclerosis and infection has been discussed by different researchers from the beginning of the nineteenth century, it is in the late seventies that the first proof of principle was obtained by Fabricant et al [78]. Specifically, this experimental study demonstrated that animals infected with the Marek virus (an avian herpes virus) showed more atherosclerotic fibroproliferative and lipid-laden lesions with respect to those untreated or fed with a high cholesterol diet, but not infected [78]. Later, *Chlamydia pneumoniae* was associated with carotid atherosclerotic damage and plaque rupture [79]. Since then, many other infectious agents, including Cytomegalovirus, *Helicobacter pylori*, herpes virus, human immunodeficiency virus, and also periodontal and influenza agents, have been suggested as possible atherosclerosis contributors, although with different strength supporting their role [79,80]. Among these pathogens it is particularly interesting the case of HCV, because this pathogen still represents one of the major globally cause of death and morbidity, with an estimated overall global prevalence of more than 184 million subjects [81]. Hepatitis C virus has been identified in atherosclerosis plaques, and may potentially act through either direct or indirect effects [82,83]. In fact, the virus has been associated to biomarkers of oxidative stress, lipid and protein oxidation, damage to DNA and mitochondrial dysfunction, and to the formation of circulating immune complexes [84]. Moreover, HCV may affect apoptosis, and heat shock protein expression [84]. It is also well known the relationship between HCV and glyco-metabolic abnormalities, from insulin resistance to diabetes [83]. Moreover, many studies have evaluated the relationship between HCV and atherosclerosis, as widely discussed in several reviews and meta-analysis. The first findings from a meta-analysis on the relationship between chronic HCV and carotid atherosclerosis, reported a higher risk for HCV infected compared to non-infected patients (OR, 95% CI: 1.76, 1.20–2.32) [85]. More recently, it has been estimated that chronic HCV infection confers a four-fold higher risk to develop carotid intimal media thickening or a carotid plaque respect to uninfected subjects [86]. The association between chronic HCV infection and the risk of CAD has been also evaluated [87,88,89]. In particular, some data evidenced that the risk of developing CAD in a person with chronic HCV is about triple the risk in uninfected persons (OR: 3.06, 95% CI: 1.99–4.72), and generally the disease is more severe [88]. Moreover, a recent systematic review and meta-analysis (297,613 HCV patients and 557,814 uninfected controls) showed a significantly increased cardio-cerebrovascular disease risk in HCV patients (OR: 1.43; 95% CI: 1.2–1.68) [89]. These findings were also confirmed when the risk of CAD (20 studies, OR: 1.382; 95% CI: 1.103–1.732) and of cerebrovascular disease (13 studies, OR: 1.48; 95% CI: 1.08–2.04) were considered separately [89]. Moreover, four studies analyzed in a meta-analysis and evaluating cardio-cerebrovascular-related deaths showed a higher risk in HCV patients than controls (OR: 1.772; 95% CI: 1.448–2.168; *p* < 0.001) [89].

Different studies found higher CV mortality in HCV infected patients [90]. In particular, a study conducted on a very large general population (23,820 adults aged 30–65 years old, follow-up 1991–2008) evidenced that HCV positive patients had higher cerebrovascular mortality (OR, 95%CI; 1.50, 1.10–2.03) [91]. Interestingly, other recent data suggested that, although HCV patients had increased risks of CV-related mortality (OR, 1.65; 95% CI, 1.07–2.56; *p* < 0.05), carotid plaques (2.27; 1.76–2.94; *p* < 0.001), and cerebro-cardiovascular events (1.30; 1.10–1.55; *p* < 0.01), the effect of HCV infection on cerebro-cardiovascular disease is even stronger in populations with a higher prevalence of diabetes (>10%) or hypertension (>20%) (1.71; 1.32–2.23; *p* < 0.001 for both) [92].

β-Thalassemia major (TM) represents a noteworthy clinical setting because TM patients are at high HCV risk to be chronically-transfused. In the last years, pharmacological and instrumental advances significantly improved survival of these patients, making cardiac disease and endocrinological complications with diabetes mellitus as major endocrinopathy, together with hepatic complications, the major sources of mortality and morbidity [83,93]. In addition, as the prevalence of HCV infection is common in these multi-transfused patients, the role of HCV in the pancreas, liver, and cardiac injury is crucial [94]. Accordingly, HCV infection appears to be involved in the pathogenesis of myocardial fibrosis through direct (myocarditis, inflammation of the heart muscle) and indirect (pancreas and liver damage with the development of T2D) mechanisms in TM patients [94]. Further data confirm the role of HCV in the induction of myocarditis and cardiomyopathies, as HCV-RNA was found in 26% of autopsied hearts from patients with hypertrophic cardiomyopathy, in 11.5% of those belonging to subjects with dilated cardiomyopathy, and 33.3% of those from patients with myocarditis [95].

In general, evidence suggested that viral eradication is associated with improvement of a number of HCV extrahepatic complications, including reduced risk of T2D and insulin resistance, improvement in myocardial perfusion defects, reduced incidence of stroke, and reduced renal and cardiovascular outcomes in T2D patients [90]. In this context, the new interferon-free therapy, which appeared more effective and better tolerated than interferon-based treatments, are of particular interest also in terms of their potential beneficial effects on CV risk, although there are not still enough data on this issue.

## 4. Psycho-Emotional Factors

Epidemiological studies have recently focused on psychosocial factors potentially increasing CV risk [96]. Accordingly, the INTERHEART study included these variables into the chart to assess CV risk, introducing the behavioral cardiology concept as a bridge between psychological factors and CV disease [97,98]. In this large multicenter study, the impact of psychological factors, including depression, locus of control, perceived stress, and experience of adverse life events, was assessed in patients with AMI. The results showed an odds ratio of 3.49 in women and 2.58 in men for the association of the psychosocial index with AMI, independent of geographic and ethnic origin [99].

Generally, psychosocial factors that induce adverse cardiac events are divided into two main categories: emotional factors, represented by affective disorders such as major depression and anxiety as well as hostility and anger, and chronic stressors including low social support, low socioeconomic status, work stress, marital stress, and caregiver strain [100]. Among emotional factors, the role of depression in promoting cardiac events in both healthy individuals and in patients with coronary artery disease has been well demonstrated [96]. Also, depression has been studied more frequently for its association with endpoints such as sudden cardiac death [101].

Recently, most epidemiological study on CV disease and psychosocial factors has focused on risk factors that operate over a long term, such as depression. Recently, considerable interest has arisen to the role that short-term risk factors, defined “triggers”, may play in CV disease onset. A trigger is considered an emotional or physical stimulus that induces physiological or pathological changes, leading to the precipitating of an acute event. In this regard, there has been increased acknowledgement of Takotsubo cardiomyopathy, which is also defined as “stress cardiomyopathy” for its relationship with acute emotional event [102].

In general, both in short and chronic psychosocial conditions, there are a number of putative mechanisms that are involved in the pathophysiological effects. A frequently postulated mechanism relating the risk of cardiac event to psychological factors is cardiac autonomic dysfunction, i.e., sympathetic predominance and parasympathetic withdrawal, but also includes alterations in inflammatory system, endothelial and platelet function, neurohormonal factors, and genetic linkages [100]. Instead, psychological factors with positive valence are associated with healthier behaviors and promote favorable physiologic effects, including improved autonomic, immune, and endothelial functions, with consequent increased well-being and enhanced resilience [103].

This is in line with a new idea of positive cardiovascular health, born from the convergence of two crucial concepts, positive health (associated with greater longevity, better quality of life, more favorable prognosis in acute illness, and improved mental health) and cardiovascular health [104]. This dimension, at individual and population levels, will examine new parameters, such as optimism and hedonic well-being that are the core focus of positive psychology, but largely neglected in preventive cardiology. According to this approach, subclinical manifestations of psychosocial distress, such as minor depression, work stress, inability to relax, may be appropriately considered in routine cardiac practice, in which next to the traditional promotion of healthy lifestyle, the acquisition and maintenance of a broader set of social and psychological factors known as protective against CV disease will be needed.

The main reasons for the cardiologists to recognize the psychosocial risk factors and manage them in the routine clinical practice are that: (i) they may trigger acute cardiac events; (ii) they are actually considered as a CV disease risk factor; (iii) they represent symptoms of cardiac disease; and (iv) they can adversely affect treatment adherence. Once evaluated, helping CV patients to initiate behavior modification is challenging, requiring skills in integrated approaches derived from psychology, and behavioral and social sciences. Moreover, this new perspective, based on multidisciplinary network, requires more intense physician monitoring and patient’s feedback before becoming more automatic and habitual.

Moreover, although different programs in management of behavioral risk factors have been studied for the ability to improve well-being and reducing risk in CV patients, there are no existing recommendations for psychosocial interventions in cardiac practice. Nonetheless, further efforts to understand values of the associations between behavioral and psychosocial factors and CV disease appear worthwhile and may lead to more widespread clinical use of new therapies to decrease this disease burden.

## 5. Conclusions

Due to the multifactorial pathogenesis of atherosclerosis, detailed risk stratification remains a complex process. There are large numbers of emerging novel biomarkers and risk factors which may fill the gap of knowledge in the CV field. The evaluation of each new parameter requires several steps, from the initial physiopathological involvement, to the prospective validation in independent cohorts, its incremental predictive value over the standard risk scores and cost-effectiveness aspects.

Clearly, in recognition of the multifactorial complexity underlying CV disease, information derived by the use of biomarkers and risk factors must be evaluated in combination with other clinical data including history, physical, and other biochemical and instrumental data. It also important to consider that for most of them a clear clinical utility, in terms of clinical-therapeutic improvement and relationship with CV risk and outcome has not yet been fully elucidated. Moreover, although there is evidence that combining biomarkers and risk factors of different nature may increase the accuracy of prediction, it is difficult to choose the optimal combinations, which may really improve the actual clinical evaluation beyond traditional utilized risk scores. Further studies evaluating whether the addition of these parameters is able to add substantial change to the predicted accuracy of traditional models are of critical importance. In this context, new biomarkers and risk factors belonging to different pathways over those traditionally included in risk algorithms, such as the recently proposed vitamin D, bone turnover biomarkers, HCV and psycho-emotional factors might be useful to better assess cardiometabolic risk. Likely, many other different or even undiscovered biomarkers may be important. Thus, in the process of new critical biomarker identification, the application of novel technologies, such as genomics, proteomics, metabolomics, and lipidomics can be particularly useful. Moreover, the application of advanced statistical approaches will permit to evaluate the real clinical relevance of each new proposed biomarker, its value at each stage of the disease, and to individuate subsets of the population that require a more accurate risk stratification.

Exploring all these possibilities and exploiting all these tools, applicability of the idea of a better biomarker-guided prevention and stratification could be practicable in a near future.
