# Erratum: Traghella, I.; et al. Nontraditional Cardiovascular Biomarkers and Risk Factors: Rationale and Future Perspectives. *Biomolecules* 2018, *8*, 40

**DOI:** 10.3390/biom8040168

**Published:** 2018-12-10

**Authors:** Irene Traghella, Francesca Mastorci, Alessia Pepe, Alessandro Pingitore, Cristina Vassalle

**Affiliations:** 1 Fondazione G. Monasterio CNR-Regione Toscana and Institute of Clinical Physiology, CNR San Cataldo Research Area, via Moruzzi, 1, 56124 Pisa, Italy; itraghella@ftgm.it (I.T.); alessia.pepe@ftgm.it (A.P.); 2 Institute of Clinical Physiology, CNR San Cataldo Research Area, via Moruzzi, 1, 56124 Pisa, Italy; mastorcif@ifc.cnr.it (F.M.); pingi@ifc.cnr.it (A.P.)

The authors wish to make the following change in their paper [1]. Due to an undetected mistake, one of the author’s name was written incorrectly. The name of Pepe Alessia is here corrected to Alessia Pepe.

The manuscript will be updated and the original will remain online on the article webpage, with a reference to this Erratum.
